# Sadness and being moved after tragic narrative exposure show opposing associations with state resilience

**DOI:** 10.3389/fpsyg.2026.1878969

**Published:** 2026-09-03

**Authors:** Rong Zhou, Yang Wu, Jinwen Zhu

**Affiliations:** College of Arts, Northeastern University, Shenyang, Liaoning, China

**Keywords:** being moved, media format, mixed emotions, narrative transportation, processing fluency, sadness, state resilience, tragic narrative

## Abstract

Tragic narrative exposure is often accompanied by mixed emotional responses, yet how specific emotional components are associated with momentary self-reported state resilience remains unclear. In a three-condition between-subjects experiment (*N* = 164), participants were assigned to a tragic animation, a tragic text, or a neutral documentary control condition. Controlling for pre-exposure resilience, baseline sadness, baseline being moved, and experimental condition, post-exposure sadness was negatively associated with post-exposure state resilience (*β* = −0.447). In contrast, post-exposure being moved showed a positive adjusted association with post-exposure state resilience in the primary model (*β* = 0.202). However, in a robustness analysis controlling for broader positive and negative affect, the association of being moved was no longer statistically significant, suggesting this positive association may partly overlap with broader positive affect. Dominance analysis indicated that post-exposure sadness showed a larger model-specific incremental contribution to explained variance than post-exposure being moved. No significant group differences emerged in post-exposure state resilience or resilience change. However, participants in both tragic narrative conditions reported higher post-exposure sadness and being moved than those in the control condition, and the animation condition showed a larger increase in being moved than the text condition. Exploratory piecewise analyses showed several significant adjacent associations among processing fluency, narrative transportation, being moved, and resilience change, but the complete serial indirect association was not supported. These exploratory results should not be interpreted as supporting a mediation model. These findings suggest that responses to tragic narrative exposure may involve mixed emotional components that are differentially associated with momentary self-reported state resilience. All results should be interpreted as adjusted associations based on same-session self-report data, rather than as causal evidence regarding resilience outcomes.

## 1 Introduction

### 1.1 Problem statement and theoretical grounding

Tragic narratives—stories centered on suffering, loss, and the inevitability of fate—are often treated as negative emotional stimuli. Yet viewers’ or readers’ responses to tragic narratives are unlikely to be uniformly negative. In response to tragic narratives, individuals may experience sadness related to characters’ suffering while simultaneously feeling moved by themes of attachment, sacrifice, moral beauty, and value affirmation. Prior research on sad films and aesthetic emotions suggests such experiences are not emotionally pure; rather, sadness and being moved often co-occur within the same episode of reception (Hanich et al., 2014; Menninghaus et al., 2015; Zickfeld et al., 2019). This shifts the central question from whether tragedy is simply harmful or beneficial to how co-occurring emotional responses after tragic narrative exposure are associated with momentary self-reported state resilience.

In the present study, state resilience refers to participants’ momentary self-reported capacity to adapt and recover under challenge. It is therefore treated as a situation-sensitive self-report construct rather than a stable personality disposition (Hiew, 2002; Ong et al., 2006). From this perspective, sadness and being moved may show different associations with state resilience after tragic narrative exposure. Drawing on Conservation of Resources theory (Hobfoll, 1989) and the process model of emotion regulation (Gross, 1998, 2015), sadness can be understood as an emotion associated with perceived loss and regulatory demand. It is often linked to loss, reduced agency, and heightened regulatory burden, and may therefore be associated with lower momentary self-reported state resilience.

Being moved, by contrast, is not a purely positive emotion, but it often involves warmth, connectedness, meaning, and the reaffirmation of core human values (Menninghaus et al., 2015; Wassiliwizky et al., 2017). These features are compatible with the broaden-and-build theory, which proposes that positive emotional experiences are associated with broader cognitive and behavioral repertoires over time (Fredrickson, 2001). Integrating this perspective with kama muta theory (Fiske et al., 2019; Seibt et al., 2017), being moved can be understood as an experience rooted in perceived intensifications of communal sharing and relational significance. Even in a tragic context, such feelings of connectedness, value affirmation, and moral elevation may be associated with higher momentary self-reported state resilience.

Taken together, these perspectives suggest that responses to tragic narratives should not be understood as simply negative. Rather, tragic narrative exposure may be accompanied by mixed emotional responses in which sadness and being moved co-occur. The critical issue is therefore not whether sadness and being moved are mutually exclusive, but whether they show distinct and potentially opposing adjusted associations with state resilience. Moreover, these associations may be asymmetrical within an acute measurement window. Because negative information is often more salient and more strongly weighted than positive information in short-term evaluations (Baumeister et al., 2001; Rozin and Royzman, 2001), sadness may show a larger model-specific contribution to explained variance in state resilience than being moved within an acute measurement window. To date, few studies have simultaneously examined the distinct and relative associations of sadness and being moved with state resilience within a single tragic narrative paradigm. In addition to this core mixed-emotion question, the present study also conducted exploratory piecewise analyses examining associations among media format, processing fluency, narrative transportation, being moved, and resilience change. These questions were examined in a three-condition experiment comparing tragic animation, tragic text, and a neutral documentary control.

### 1.2 Literature review

#### 1.2.1 Resilience and emotional experience

Psychological resilience generally refers to the capacity to maintain, recover, or rebuild adequate psychological functioning in the face of adversity, stress, or trauma (Masten, 2001; Luthar et al., 2000). The present study focuses specifically on state resilience, which emphasizes an individual’s momentary self-reported evaluation of their capacity to adapt and recover within a given situation rather than a stable personality disposition. Because state resilience is treated as more situation-sensitive than trait resilience (Hiew, 2002; Ong et al., 2006), it is a relevant self-report construct for examining momentary self-evaluations after narrative exposure.

Prior research suggests that positive emotions are often associated with resilience-related outcomes and broader cognitive and behavioral repertoires (Fredrickson, 2001; Fredrickson et al., 2003), whereas resilience more broadly reflects the capacity to maintain or recover psychological functioning in the face of adversity (Bonanno, 2004). Intense negative emotions, meanwhile, are often discussed in relation to regulatory demands and lower self-evaluations of adaptive capacity (Hobfoll, 1989).

This divergence suggests that the associations between emotion and state resilience may not be adequately captured by a simple positive–negative dichotomy. Instead, it is useful to distinguish among specific emotion types and their experiential features. In the context of tragic narratives, emotional responses are rarely uniformly negative and may simultaneously include sadness, being moved, and other components. Examining how tragic narrative experience is associated with state resilience therefore requires attention to the distinct associations of specific emotional components rather than merely assessing whether negative affect is present.

#### 1.2.2 Sadness and being moved: two core emotions in tragic narratives

Sadness is a prototypical emotional response to tragic narratives and is typically associated with plot elements involving characters’ suffering, loss, separation, or loss of control over fate. Theoretically, sadness is associated with feelings of loss, helplessness, and reduced agency, and may be accompanied by increased emotion regulation demands in the short term (Gross, 2015; Hobfoll, 1989). In acute tragic contexts, sadness may therefore be associated with lower momentary self-reported state resilience.

Being moved, by contrast, is a more complex emotional experience. Research has shown that being moved is not simply a positive emotion but is often accompanied by moist eyes, a lump in the throat, feelings of warmth, and a strong sense of meaning, and is often conceptualized as a mixed-valence aesthetic emotion (Menninghaus et al., 2015; Wassiliwizky et al., 2017). Two prominent theoretical accounts are relevant to its experiential structure. First, kama muta theory conceptualizes being moved as related to the perception of a sudden intensification of communal sharing relationships, an experience associated with connectedness, prosocial motivation, and belonging (Fiske et al., 2019; Seibt et al., 2017; Zickfeld et al., 2019). Second, the appraisal model of being moved emphasizes the relevance of attachment relationships, prosocial values, moral beauty, and meaning-making processes to this emotional experience (Menninghaus et al., 2015).

Tragic narratives are often accompanied by both sadness and a sense of being moved. While characters’ suffering and loss may be associated with sadness, the depicted attachment, sacrifice, and commitment between characters may be associated with a feeling of being moved. Thus, tragic experience is not merely a negative emotional event but a complex situation with multiple emotional components (Larsen and McGraw, 2011; Berrios et al., 2015). Within this context, sadness and being moved are not mutually exclusive; they can coexist as core components, each with different associations with state resilience.

However, existing research typically examines sadness or being moved in isolation, rarely integrating both into a single analytical framework to compare their distinct associations with the same self-reported outcome. For example, studies on appreciation and meaningful entertainment have emphasized reflective or eudaimonic responses without directly modeling the potentially divergent associations of sadness and being moved (Oliver and Bartsch, 2010; Oliver and Raney, 2011). Similarly, research on sad films has primarily focused on why audiences value such experiences, rather than on how specific emotional components are associated with self-reported outcomes such as state resilience (Hanich et al., 2014). Consequently, whether sadness and being moved show distinct and potentially opposing adjusted associations within an acute measurement window remains underexamined.

#### 1.2.3 Media format and aesthetic experience

Beyond the emotional components, the experience of a tragic narrative can also vary by media format. Differences in how media encode information, engage sensory channels, and demand processing may be associated with how individuals receive narratives, as well as their reported aesthetic experience and emotional responses.

Processing fluency refers to the subjective ease with which individuals perceive and comprehend information (Reber et al., 2004). Research indicates that higher processing fluency is often associated with more positive evaluations and smoother aesthetic experiences (Graf and Landwehr, 2015). Theoretically, visual media such as animation can simultaneously deliver images, sound, movement, and facial expressions across multiple sensory channels, and may be associated with lower comprehension burden and higher subjective fluency under certain conditions. However, the consistent appearance of such fluency differences may depend on material characteristics, presentation duration, and individual processing styles.

Narrative transportation refers to a psychological state in which individuals become cognitively, emotionally, and imaginatively absorbed into a story world during narrative exposure (Green and Brock, 2000). High narrative transportation is characterized by temporary disengagement from immediate reality and deeper immersion in the narrative. Research has shown that narrative transportation is associated with stronger emotional responses and may also be associated with attitudes, beliefs, and prosocial tendencies (Green and Brock, 2000; van Laer et al., 2014; Appel et al., 2015).

Based on these findings, media format may be associated with reported tragic narrative experience not only at the group level but also in relation to aesthetic experience variables. Specifically, different media formats may be associated with processing fluency, narrative transportation, and complex emotional experiences such as being moved. However, whether these variables are associated with state resilience in tragic narrative contexts remains uncertain, particularly given the limited empirical work examining media format, aesthetic experience, emotional response, and state resilience within a single framework. The present study therefore adopted an exploratory piecewise approach to examining these associations.

### 1.3 Hypotheses

Based on the theoretical analysis above, the present study conceptualized responses to tragic narratives as involving mixed emotional components, including sadness and being moved, and examined whether these two post-exposure emotional responses showed opposing adjusted associations with state resilience. The following hypotheses and exploratory question were examined:

*H1*: Controlling for pre-exposure resilience, baseline emotion levels, and experimental condition, post-exposure sadness would be negatively associated with post-exposure state resilience.

*H2*: Controlling for pre-exposure resilience, baseline emotion levels, and experimental condition, post-exposure being moved would be positively associated with post-exposure state resilience.

*H3*: When both post-exposure sadness and post-exposure being moved were entered simultaneously with pre-exposure resilience, baseline emotion levels, and experimental condition controlled, post-exposure sadness would show a larger model-specific incremental contribution to explained variance in post-exposure state resilience than post-exposure being moved within the acute measurement window.

*H4 (manipulation check)*: The tragic narrative groups (animation and text) were expected to report higher post-exposure sadness and being moved than the documentary control group.

*H4a*: The animation group was expected to show a larger reported increase in being moved than the text group.

Exploratory analysis: In the tragic narrative subsample, we conducted exploratory piecewise analyses to examine whether processing fluency, narrative transportation, being moved, and resilience change were associated with one another.

## 2 Materials and methods

### 2.1 Participants

Adult participants were recruited from universities and communities through the Credamo (见数) online survey platform, yielding 200 completed questionnaires. An *a priori* power analysis indicated that a minimum sample of 159 participants was required to detect a medium effect size (*f*^2^ = 0.10) in a multiple regression with seven predictors at *α* = 0.05 and 80% power (G*Power; Faul et al., 2009). The final analytic sample of 164 participants exceeded this threshold. Data were screened according to the following eligibility criteria: (1) age between 18 and 45 years; (2) normal or corrected-to-normal vision and normal hearing; (3) no reading difficulties and ability to fluently comprehend the study materials; (4) no history of psychiatric or neurological disorders and no current use of psychotropic medication; (5) no indication of aphantasia based on two visual imagery screening items; and (6) successful completion of the embedded attention check. Of the 200 participants initially recruited, 36 were excluded because they reported psychiatric or neurological conditions, failed the visual imagery screening, or failed the embedded attention check. The final analytic sample comprised 164 participants (valid response rate: 82.0%), with 53 in the animation group, 54 in the text group, and 57 in the documentary control group. Participants were randomly assigned to one of the three conditions using the randomization function of Credamo.

### 2.2 Design

The study employed a single-factor, three-level between-subjects design, with experimental condition (tragic animation, tragic text, documentary control) as the between-subjects factor. The primary self-report outcome was post-exposure state resilience. Sadness and being moved were measured both pre- and post-exposure as self-reported focal emotional responses. Processing fluency and narrative transportation were included as aesthetic experience variables in the exploratory analysis.

### 2.3 Materials

The animation group viewed Disney’s animated short film adaptation of The Little Matchgirl (2006; approximately 6–7 min), an Academy Award-nominated animated short directed by Roger Allers. The film depicts a child’s suffering, sacrifice, and emotional bond with her grandmother. The text group read a Chinese text-based narrative adapted from Ye Junjian’s widely circulated Chinese translation of Andersen’s The Little Match Girl. The text was edited to match the animation’s core plot and event sequence as closely as possible. The text stimulus contained approximately 3,272 Chinese characters. The documentary control group viewed a neutral documentary about match production (approximately 5 min), selected to provide a non-tragic comparison with minimal tragic emotional content. The documentary control condition was intended to provide a neutral, non-tragic exposure, although it was not a fully matched neutral fictional narrative control. The three materials were approximately matched in exposure duration to reduce potential confounding by viewing or reading time. All materials were delivered via Credamo. Data collection took place between 1 February 2026 and 14 February 2026. No separate pilot study was conducted to validate the reported emotional response profiles of the materials prior to the main experiment; this issue is revisited in the Limitations section.

### 2.4 Measures

All measures were administered in Chinese. The State Resilience Scale, I-PANAS-SF, Processing Fluency Scale, Narrative Transportation Scale, and single-item emotion measures were translated or adapted into Chinese for the present study. The research team reviewed these translations for semantic clarity and conceptual consistency with the original constructs. No formal back-translation procedure was conducted; this should be considered when interpreting findings based on translated or adapted measures. The Chinese wording of the single-item sadness measure was “你此刻在多大程度上感到悲伤?” and the Chinese wording of the single-item being moved measure was “你此刻在多大程度上感到被感动?” The term “被感动” is a commonly used Chinese expression referring to feeling moved or touched by an emotionally meaningful event. Because this wording may encompass related nuances such as being moved, touched, or emotionally stirred, the findings related to being moved should be interpreted with this linguistic scope in mind.

#### 2.4.1 State resilience scale

State resilience was measured using the 15-item self-report State Resilience Scale (SRS; Hiew, 2002), rated on a 5-point Likert scale (1 = completely disagree, 5 = completely agree). Higher scores indicate greater momentary state resilience (pre-exposure *α* = 0.903; post-exposure α = 0.947). The scale assessed participants’ momentary self-reported state resilience rather than stable trait resilience.

#### 2.4.2 Emotion measures

The International Short-Form Positive and Negative Affect Schedule (I-PANAS-SF; Thompson, 2007) was administered, comprising 10 items across positive and negative affect subscales. In this study, the I-PANAS-SF was included to characterize the broader affective context of the exposure session, while hypothesis-driven analyses focused on the focal sadness and being moved items. Sadness was assessed with the item “To what extent do you feel sad right now?” The Chinese wording was “你此刻在多大程度上感到悲伤?” Being moved was assessed with the item “To what extent do you feel moved right now?” The Chinese wording was “你此刻在多大程度上感到被感动?” Both items were rated on a 5-point Likert scale (1 = very slightly or not at all, 5 = extremely). Given the pre-post repeated-measures design and focus on momentary emotional intensity, single-item ratings were used to reduce participant burden and response fatigue. However, this approach cannot assess the multidimensional structure or internal consistency of sadness or being moved and may be sensitive to linguistic nuances in the Chinese emotion labels, especially for “被感动” (Larsen and Fredrickson, 1999).

#### 2.4.3 Processing fluency scale

Processing fluency was measured using the four-item perceptual fluency scale developed by Graf et al. (2018), rated on a 5-point Likert scale (1 = very difficult, 5 = very easy). Internal consistency in the present sample was *α* = 0.640. Given the small number of items, this relatively low coefficient should be considered when interpreting results involving this measure.

#### 2.4.4 Narrative transportation scale

Narrative transportation was measured using an 11-item transportation scale adapted from Green and Brock (2000), with items rated on a 5-point Likert scale (1 = completely disagree, 5 = completely agree). This scale was administered only in the animation and text groups (*n =* 107). Item 5 was reverse-scored. Internal consistency in the present sample was α = 0.505. The original scale was developed for longer narrative contexts, and the brief narrative stimuli used in the present study (approximately 6 min) may have reduced item discriminability and internal consistency. Additionally, variability in attention and device conditions in the online testing environment may have introduced additional measurement error. Results involving narrative transportation should therefore be interpreted with these measurement limitations in mind. Accordingly, analyses involving narrative transportation were treated as exploratory.

### 2.5 Procedure

The experiment was conducted online using Credamo. Participants were instructed to complete the study on their personal devices in a quiet environment and to minimize distractions during stimulus exposure. The procedure was as follows: (1) participants provided electronic informed consent and completed demographic information and screening items; (2) participants completed pre-exposure measures, including the State Resilience Scale and emotion measures; (3) based on the random assignment generated by Credamo, the animation group viewed the tragic animated film, the text group read the matched written story, and the documentary control group viewed the neutral documentary; (4) immediately after exposure, participants completed the processing fluency scale (all three groups) and the narrative transportation scale (animation and text groups only); (5) participants completed post-exposure measures, including the State Resilience Scale and emotion measures; (6) participants completed an embedded attention check and content-related compliance items designed to verify that they had attended to the assigned material; and (7) participants were debriefed and thanked. The entire procedure took approximately 15–20 min.

### 2.6 Analytic strategy

Data analysis proceeded in the following steps. First, descriptive statistics and scale reliabilities were reported. Second, Pearson correlations were computed among key variables. Third, one-way analyses of variance (ANOVAs) examined group differences in baseline variables, post-exposure variables, and change scores, with Tukey HSD *post-hoc* comparisons. Fourth, multiple regression analysis examined the adjusted associations of post-exposure sadness and post-exposure being moved with post-exposure state resilience, controlling for pre-exposure resilience, baseline emotions, and experimental condition. Because sadness and being moved were measured rather than experimentally manipulated, these regression models were interpreted as association-based analyses rather than causal tests. Dominance analysis was used to compare the model-specific incremental explanatory contributions of post-exposure sadness and post-exposure being moved beyond the control variables. To assess robustness, HC3 heteroscedasticity-consistent standard errors and a sensitivity analysis excluding influential cases based on Cook’s distance were also conducted. An additional robustness model including broader positive and negative affect scores from the I-PANAS-SF was conducted to examine whether the focal associations remained after accounting for broader affective states. Finally, in the tragic narrative subsample (animation and text groups, *n* = 107), exploratory piecewise regression analyses examined the associations among media format, processing fluency, narrative transportation, post-exposure being moved, and resilience change. Given the modest subsample size and the low reliability of some exploratory measures, these analyses were interpreted as exploratory piecewise associations rather than as evidence for a supported mediation model or a confirmatory structural equation model. All analyses were conducted using R.

## 3 Results

### 3.1 Descriptive statistics

Table 1 presents descriptive statistics for key variables across the full sample. Descriptively, sadness and being moved increased from pre- to post-exposure (sadness change M = 0.69, SD = 1.31; being moved change M = 0.75, SD = 1.23), whereas mean state resilience showed a small descriptive decrease (resilience change M = −0.07, SD = 0.53). Processing fluency was above the scale midpoint (M = 4.37, SD = 0.46), and narrative transportation in the two tragic narrative groups was also relatively high (M = 4.22, SD = 0.30).

**Table 1:** Descriptive statistics for key variables.

Variable	*N*	M	SD	Min	Max
Pre-exposure resilience	164	4.20	0.50	1.93	4.93
Post-exposure resilience	164	4.13	0.65	1.53	5.00
Resilience change (post − pre)	164	−0.07	0.53	−2.93	1.67
Pre-exposure sadness	164	1.51	0.79	1.00	5.00
Post-exposure sadness	164	2.20	1.22	1.00	5.00
Sadness change (post − pre)	164	0.69	1.31	−3.00	4.00
Pre-exposure being moved	164	2.75	1.27	1.00	5.00
Post-exposure being moved	164	3.50	1.22	1.00	5.00
Being moved change (post − pre)	164	0.75	1.23	−2.00	4.00
Processing fluency	164	4.37	0.46	1.50	5.00
Narrative transportation	107	4.22	0.30	3.09	4.82

Narrative transportation was measured only in the animation and text groups. State Resilience Scale: pre-exposure *α* = 0.903, post-exposure *α* = 0.947; processing fluency *α* = 0.640; narrative transportation *α* = 0.505. Group-level descriptive statistics are presented in Table 3.

### 3.2 Correlation analysis

Table 2 presents Pearson correlation coefficients among key variables. Post-exposure sadness was significantly negatively correlated with post-exposure state resilience (*r* = −0.441, *p* < 0.001), whereas post-exposure being moved was significantly positively correlated with post-exposure state resilience (*r* = 0.360, *p* < 0.001). These zero-order correlations were consistent with the directions examined in the regression analyses. Pre-exposure state resilience was strongly correlated with post-exposure state resilience (*r* = 0.612, *p* < 0.001), supporting the inclusion of baseline resilience as a covariate in subsequent analyses. Notably, the zero-order correlation between pre-exposure sadness and post-exposure resilience was non-significant (*r* = −0.043, *p* = 0.583), whereas pre-exposure sadness was significantly positively correlated with resilience change (*r* = 0.236, *p* < 0.01). This pattern may reflect a possible suppression effect, which was considered in the interpretation of the regression results.

**Table 2:** Correlation matrix for key variables (*N =* 164).

Variable	1	2	3	4	5	6	7	8
1. Pre-exposure resilience	—							
2. Post-exposure resilience	0.612***	—						
3. Pre-exposure sadness	−0.306***	−0.043	—					
4. Post-exposure sadness	−0.142	−0.441***	0.202**	—				
5. Pre-exposure being moved	0.328***	0.388***	0.210**	−0.186*	—			
6. Post-exposure being moved	0.270***	0.360***	0.162*	0.079	0.517***	—		
7. Resilience change	−0.182*	0.666***	0.236**	−0.414***	0.173*	0.193*	—	
8. Processing fluency	0.212**	0.263***	−0.073	−0.040	−0.042	0.136	0.126	—

*p < 0.05, **p < 0.01, ***p < 0.001.

### 3.3 Group comparisons

#### 3.3.1 Baseline equivalence

One-way ANOVAs indicated no statistically significant group differences in pre-exposure state resilience (*F*(2, 161) = 0.547, *p =* 0.580, η^2^ = 0.007), pre-exposure sadness (*F*(2, 161) = 2.870, *p =* 0.060, η^2^ = 0.034), or pre-exposure being moved (*F*(2, 161) = 1.669, *p =* 0.192, η^2^ = 0.020), suggesting no statistically detectable baseline differences among the three groups on these variables.

#### 3.3.2 Post-exposure emotions and resilience

Post-exposure sadness differed significantly across groups (*F*(2, 161) = 28.323, *p <* 0.001, η^2^ = 0.260). Both the animation and text groups were significantly higher than the documentary control group but did not differ from each other. Post-exposure being moved also differed significantly across groups (*F*(2, 161) = 11.034, *p <* 0.001, η^2^ = 0.121). Participants in both tragic narrative groups reported significantly higher being moved than those in the control group. For being moved change, the animation group scored significantly higher than the text group (Tukey *p =* 0.025), corresponding to a larger observed increase in self-reported being moved in the animation condition than in the text condition (see Table 3).

**Table 3:** Group means, standard deviations, and ANOVA results for key variables.

Variable	Animation M (SD)	Text M (SD)	Control M (SD)	*F*	*p*	η^2^
Pre-exposure resilience	4.22 (0.44)	4.15 (0.55)	4.24 (0.49)	0.547	0.580	0.007
Post-exposure resilience	4.14 (0.52)	4.03 (0.78)	4.22 (0.63)	1.159	0.316	0.014
Resilience change	−0.08 (0.52)	−0.12 (0.65)	−0.02 (0.38)	0.448	0.640	0.006
Pre-exposure sadness	1.55 (0.77)	1.67 (1.03)	1.32 (0.47)	2.870	0.060	0.034
Post-exposure sadness	2.57 (1.12)a	2.72 (1.35)a	1.35 (0.55)b	28.323	<0.001	0.260
Sadness change	1.02 (1.31)a	1.06 (1.56)a	0.04 (0.68)b	12.351	<0.001	0.133
Pre-exposure being moved	2.55 (1.29)	2.70 (1.31)	2.98 (1.20)	1.669	0.192	0.020
Post-exposure being moved	3.98 (0.99)a	3.59 (1.11)a	2.96 (1.31)b	11.034	<0.001	0.121
Being moved change	1.43 (1.20)a	0.89 (1.24)b	−0.02 (0.72)c	25.807	<0.001	0.243
Processing fluency	4.44 (0.27)	4.39 (0.35)	4.27 (0.65)	2.148	0.120	0.026

Post-hoc comparisons used Tukey HSD. For post-exposure sadness and sadness change, animation = text > control (ps < 0.001). For post-exposure being moved, animation = text > control (ps < 0.01). For being moved change, animation > text (p = 0.025), and both animation and text > control (ps < 0.001). Shared superscript letters indicate non-significant pairwise differences.

The three groups did not differ significantly in post-exposure state resilience (*F*(2, 161) = 1.159, *p* = 0.316) or resilience change (*F*(2, 161) = 0.448, *p* = 0.640), indicating no statistically significant group-level differences in self-reported state resilience were detected in the present sample. Processing fluency also did not differ significantly across groups (*F*(2, 161) = 2.148, *p* = 0.120). Within the two tragic narrative groups, narrative transportation did not differ significantly between the animation and text conditions (*t*(105) = 0.917, *p* = 0.361).

### 3.4 Primary regression model: adjusted associations of sadness and being moved with state resilience

Post-exposure state resilience was regressed on pre-exposure resilience, pre-exposure sadness, pre-exposure being moved, group dummy variables (with documentary control as the reference), post-exposure sadness, and post-exposure being moved. To assess robustness, HC3 heteroscedasticity-consistent standard errors (Model 2) and a sensitivity analysis excluding influential cases based on Cook’s distance (Model 3) were also conducted.

The overall model was statistically significant (*F*(7, 156) = 32.28, *p <* 0.001) and accounted for 59.2% of the variance in post-exposure state resilience. The adjusted *R*^2^ was 0.574. Controlling for pre-exposure resilience, pre-exposure sadness, pre-exposure being moved, and experimental condition, post-exposure sadness was significantly negatively associated with post-exposure state resilience (*β* = −0.447, *p <* 0.001), whereas post-exposure being moved was significantly positively associated with post-exposure state resilience (*β* = 0.202, *p =* 0.004). In the primary model, these results were consistent with H1 and H2 (see Table 4).

**Table 4:** Adjusted regression models predicting post-exposure state resilience.

Predictor	Model 1 B (SE)	*β*	Model 2 B (SE)	*β*	Model 3 B (SE)	*β*
Pre-exposure resilience	0.731 (0.079)	0.555	0.731 (0.097)	0.555	0.748 (0.061)	0.711
Pre-exposure sadness	0.144 (0.049)	0.176	0.144 (0.049)	0.176	0.110 (0.024)	0.196
Pre-exposure being moved	−0.005 (0.036)	−0.010	−0.005 (0.039)	−0.010	−0.019 (0.020)	−0.053
Post-exposure sadness	−0.240 (0.033)	−0.447	−0.240 (0.054)	−0.447	−0.117 (0.023)	−0.296
Post-exposure being moved	0.109 (0.037)	0.202	0.109 (0.049)	0.202	0.090 (0.025)	0.246
Group (text vs. control)	0.090 (0.097)	0.065	0.090 (0.078)	0.065	0.028 (0.048)	0.030
Group (animation vs. control)	0.083 (0.102)	0.060	0.083 (0.092)	0.060	−0.048 (0.053)	−0.051
*N*	164		164		152	
*R* ^2^	0.592		0.592		0.700	

Dependent variable: post-exposure state resilience. Reference group: documentary control. Model 2 uses HC3 heteroscedasticity-consistent standard errors. Model 3 excludes 12 influential cases identified based on Cook’s distance exceeding 4/N. All VIFs <2.5. In Model 2, HC3 estimation adjusts standard errors only; point estimates remain identical to OLS.

In terms of standardized coefficients, the coefficient for post-exposure sadness was larger in absolute magnitude than that for post-exposure being moved (|*β*| = 0.447 vs. 0.202). Dominance analysis (see Section 3.4.1) further indicated that, within this primary model, post-exposure sadness showed a larger model-specific incremental contribution to explained variance than post-exposure being moved, consistent with H3.

Neither group dummy variable was significant in any model, suggesting no significant adjusted association between experimental condition and post-exposure state resilience after accounting for the emotion variables. Pre-exposure being moved was non-significant after post-exposure emotions were entered (*β* = −0.010, *p =* 0.889). Pre-exposure sadness showed a significant positive coefficient (*β* = 0.176, *p =* 0.004), which is discussed further in Section 4.1.

Under HC3 robust standard errors (Model 2), both post-exposure sadness (*p <* 0.001) and post-exposure being moved (*p =* 0.028) remained significant, with unchanged coefficient directions. After excluding 12 influential cases identified by Cook’s distance exceeding 4/N (Model 3, *n =* 152), post-exposure sadness (β = −0.296, *p <* 0.001) and post-exposure being moved (*β* = 0.246, *p <* 0.001) maintained consistent directions and significance patterns. The flagged cases were distributed across all three groups, and their removal did not alter the overall pattern of coefficient directions or statistical significance.

As an additional robustness check, broader positive and negative affect scores from the I-PANAS-SF were entered into the regression model. After accounting for broader affective states, post-exposure sadness remained negatively associated with post-exposure state resilience (*β* = −0.137, *p =* 0.010). Post-exposure being moved remained positively associated with post-exposure state resilience, but this association no longer reached conventional significance (*β* = 0.089, *p =* 0.101). Post-exposure positive affect was positively associated with post-exposure state resilience (*β* = 0.338, *p <* 0.001), whereas post-exposure negative affect was negatively associated with post-exposure state resilience (*β* = −0.444, *p <* 0.001). These results suggest that the negative association of sadness was more robust to adjustment for broader affective states, whereas the positive association of being moved was less robust and may partly overlap with broader positive affect.

#### 3.4.1 Dominance analysis

Within the primary regression model, dominance analysis was conducted to compare the incremental explanatory contributions of post-exposure sadness and post-exposure being moved to the explained variance in post-exposure state resilience, beyond the control variables (experimental condition, pre-exposure resilience, pre-exposure sadness, and pre-exposure being moved).

After controlling for experimental condition, pre-exposure resilience, pre-exposure sadness, and pre-exposure being moved, the general dominance weight for post-exposure sadness was 0.1412 and that for post-exposure being moved was 0.0225. Within this primary model, post-exposure sadness showed a larger incremental contribution to explained variance in post-exposure state resilience than post-exposure being moved, consistent with H3 (see Table 5).

**Table 5:** Model-specific incremental variance contributions of post-exposure sadness and being moved.

Indicator	Post-exposure sadness	Post-exposure being moved
ΔR^2^ when entered alone beyond controls	0.1415	0.0228
ΔR^2^ when entered after the other post-exposure emotion	0.1408	0.0221
General dominance weight (average incremental R^2^)	0.1412	0.0225
Proportion of incremental variance explained by the two post-exposure emotions	86.3%	13.7%

Control variable model *R*^2^ = 0.428. Full model (controls + both post-exposure emotions) *R*^2^ = 0.592. Higher general dominance weights indicate larger model-specific incremental contributions to explained variance.

Importantly, this incremental contribution should not be interpreted as psychological priority, causal strength, or evidence that sadness is more consequential than being moved; it may also reflect differences in measurement precision, variability, salience, or conceptual proximity to self-reported state resilience.

### 3.5 Supplementary analysis: resilience change and emotion change

As a supplementary check of the main regression pattern, additional regression analyses were conducted using resilience change (post − pre) as the dependent variable.

When the dependent variable was replaced with resilience change, the coefficient directions of post-exposure sadness and post-exposure being moved were consistent with the primary model (Model A). When emotion change scores were used directly (Model B), sadness change showed a significant negative coefficient for resilience change (*β* = −0.599, *p <* 0.001), whereas being moved change showed a positive coefficient that did not reach conventional significance (*β* = 0.145, *p =* 0.059). This pattern may reflect the accumulation of measurement error inherent in difference scores. Overall, the supplementary analyses showed a pattern broadly consistent in direction with the primary regression results, although the findings based on change scores should be interpreted cautiously (see Table 6).

**Table 6:** Supplementary adjusted regression models predicting resilience-change scores.

Model	Key predictor	*B*	*β*	*t*	*p*
Model A: Pre/post emotions + group	Pre-exposure sadness	0.211	0.319	4.572	<0.001
	Post-exposure sadness	−0.238	−0.551	−7.038	<0.001
	Post-exposure being moved	0.092	0.212	2.400	0.018
Model B: Emotion change + group	Sadness change	−0.240	−0.599	−8.375	<0.001
	Being moved change	0.062	0.145	1.900	0.059

Model A: dependent variable = resilience change; variables entered into the model include pre- and post-exposure sadness, pre- and post-exposure being moved, and experimental condition. Model B: dependent variable = resilience change; variables entered into the model include sadness change, being moved change, and experimental condition.

### 3.6 Exploratory piecewise analysis: aesthetic experience, emotional response, and resilience change

In the tragic narrative subsample (animation and text groups, *n* = 107), exploratory piecewise regression analyses examined associations among media format, processing fluency, narrative transportation, being moved, and resilience change. Media format was coded as animation = 1 and text = 0, and separate regression models were estimated for each adjacent association in this exploratory piecewise analysis.

The exploratory piecewise analysis showed that processing fluency was significantly positively associated with narrative transportation (*B* = 0.354, *p* < 0.001), narrative transportation was significantly positively associated with post-exposure being moved (*B* = 0.885, *p* = 0.003), and post-exposure being moved was significantly positively associated with resilience-change scores (*B* = 0.159, *p* = 0.004). However, media format was not significantly associated with processing fluency (*B* = 0.050, *p* = 0.409). These results suggest that several adjacent associations in the exploratory piecewise analysis were statistically significant, whereas the association between media format and processing fluency was not (see Table 7).

**Table 7:** Exploratory piecewise regression coefficients in the tragic narrative subsample.

Path	*B*	SE	*t*	*p*
a: Media format → Processing fluency	0.050	0.060	0.829	0.409
b: Processing fluency → Narrative transportation	0.354	0.088	4.013	<0.001
c: Narrative transportation → Post-exposure being moved	0.885	0.289	3.060	0.003
d: Post-exposure being moved → Resilience change	0.159	0.054	2.951	0.004

Model B controls for media format. Model C controls for media format and pre-exposure being moved. Model D controls for media format, processing fluency, narrative transportation, and pre-exposure resilience.

Supplementary bootstrap analyses did not support a complete serial indirect association with resilience change (estimate = −0.182, 95% CI [−1.037, 0.385]). Therefore, the exploratory analyses showed several significant pairwise associations among aesthetic experience, emotional response, and resilience change, but did not support a complete serial indirect association.

## 4 Discussion

### 4.1 Opposing associations of sadness and being moved with state resilience

The central finding of this study is that sadness and being moved, as co-occurring self-reported emotional responses after tragic narrative exposure, showed opposing adjusted associations with post-exposure state resilience. Post-exposure sadness was significantly negatively associated with post-exposure state resilience (*β* = −0.447), whereas post-exposure being moved showed a significant positive association (*β* = 0.202). Both coefficients remained significant after controlling for pre-exposure resilience, baseline emotions, and experimental condition. This result is consistent with the core theoretical premise of the study: tragic experience is not a unidirectionally negative event but a mixed-emotion context in which different emotional components may be differentially linked to momentary self-reported state resilience.

Post-exposure sadness showed the largest negative standardized coefficient for post-exposure state resilience among the focal post-exposure emotion variables, and this pattern remained stable across HC3 robust standard errors and the influential-case sensitivity analysis. This pattern may be interpreted in light of Conservation of Resources theory, which suggests that sadness may be associated with perceived loss, reduced agency, and greater regulatory demand. From the perspective of the process model of emotion regulation, the pain and loss associated with tragic narratives may be accompanied by greater regulatory demand, which may be linked to lower momentary self-reported state resilience within a short measurement window.

Post-exposure being moved showed a positive adjusted association with post-exposure state resilience in the primary model after controlling for multiple baseline variables, and this association remained evident in the influential-case sensitivity analysis. This pattern suggests that being moved is not reducible to sadness within tragic experience, but may represent a distinct emotional component associated with momentary self-reported state resilience. This pattern can be discussed alongside the broaden-and-build theory, which proposes that positive emotional experiences broaden momentary cognitive-behavioral repertoires and contribute to adaptive resources over time. Kama muta theory provides a more specific interpretive framework: being moved is conceptualized as related to the perception of a sudden intensification of communal sharing relationships and is associated with connectedness, prosocial motivation, and a sense of meaning. In the tragic context, being moved may therefore be better understood as a distinct component of the mixed emotional response rather than as a simple positive “add-on” to sadness. However, when broader positive and negative affect scores were additionally controlled, the association between post-exposure being moved and post-exposure state resilience remained positive in direction but no longer reached conventional significance. This suggests that the being moved association should be interpreted more cautiously and may partly overlap with broader positive affect.

Notably, the dominance analysis indicated that, after controlling for experimental condition, pre-exposure resilience, pre-exposure sadness, and pre-exposure being moved, post-exposure sadness contributed more incrementally to the explained variance in post-exposure state resilience than post-exposure being moved. This statistical asymmetry may be considered alongside the classic “bad is stronger than good” proposition (Baumeister et al., 2001; Vaish et al., 2008), which suggests that negative experiences can have high processing priority and immediate salience. At the same time, alternative explanations should be noted: sadness may have been more readily captured than being moved by a single-item measure, and the two variables may have differed in measurement precision, variability, or conceptual proximity to self-reported state resilience. More generally, the present study did not directly measure resource-related processes; therefore, any interpretation in terms of resource loss or adaptive resource development should be treated as a theoretical possibility rather than as a conclusion supported by the present data. This does not negate the relevance of being moved; rather, it suggests that its positive adjusted association with state resilience was less robust than the negative adjusted association of sadness within this same-session self-report model.

Additionally, pre-exposure sadness showed a significant positive coefficient in the final model (*β* = 0.176, *p =* 0.004). This seemingly counterintuitive result may be attributable to a statistical suppression effect: pre-exposure sadness was negatively correlated with pre-exposure state resilience (*r =* −0.306), but after controlling for pre-exposure resilience, the direction of its partial association with post-exposure resilience reversed. In other words, the positive coefficient of pre-exposure sadness in the model should not be directly interpreted as “sadness is inherently beneficial” but may reflect a change in the partial coefficient when multiple correlated predictors enter the model simultaneously. This coefficient is therefore better treated as a supplementary statistical observation rather than as a core theoretical conclusion, and it requires further verification in future research.

### 4.2 Interpreting non-significant group differences

The three groups did not differ significantly in post-exposure state resilience or resilience change, and this pattern was consistent across analyses. This null pattern should not be interpreted as evidence that media format is irrelevant; rather, it indicates that statistically significant group-level differences in self-reported state resilience were not detected in the present sample. Although animation and text differ in sensory channels and information processing demands, the present data suggest some similarity in participants’ reported sadness and being moved within this tragic narrative context. At the same time, this null group pattern should be interpreted cautiously because the observed η^2^ for post-exposure resilience was small, and the study may not have been optimally powered to detect subtle between-group differences in resilience.

Notably, although the three groups did not differ significantly in post-exposure state resilience, they did differ significantly in post-exposure sadness and being moved. Participants in both the animation and text groups reported significantly higher post-exposure sadness and being moved than those in the documentary control group, and the animation group showed a larger observed increase in self-reported being moved than the text group. This pattern suggests that the tragic narrative conditions were associated with higher reported levels of sadness and being moved than the documentary control condition, whereas statistically significant group-level differences in self-reported state resilience were not observed. The larger observed increase in self-reported being moved in the animation condition may be related to the simultaneous availability of facial expressions, body movements, color, and music, which may be associated with stronger reported empathic or relational engagement.

### 4.3 Exploratory piecewise associations and their limitations

The present study offers three theoretical contributions. First, the study highlights the mixed emotional structure of responses to tragic narratives. Existing research has typically examined sadness and being moved separately, rarely comparing their relative statistical associations within a single model. By simultaneously entering post-exposure sadness and post-exposure being moved into the same regression model and further conducting dominance analysis, the present study showed that both were oppositely associated with self-reported state resilience in the primary model, and that post-exposure sadness showed a larger model-specific incremental contribution to explained variance than post-exposure being moved. This suggests that the self-reported correlates of tragic narrative exposure cannot be reduced to a single negative emotional response but should be understood in relation to multiple co-occurring emotional components.

These results suggest a tentative pattern of adjacent associations: higher processing fluency was associated with stronger narrative transportation, stronger narrative transportation was associated with greater post-exposure being moved, and greater post-exposure being moved was associated with higher resilience-change scores. At the same time, media format itself was not significantly associated with processing fluency, and the complete serial indirect association was not supported. Therefore, these exploratory results should not be interpreted as supporting a mediation model.

Several factors may help explain the non-significant association between media format and processing fluency. First, the text and animation materials in this study were closely matched in core plot and informational content, which may have reduced differences in subjective fluency between the two formats. Second, individual differences in device conditions and attention states in the online testing environment may have reduced the detectability of fluency differences associated with media format. Regarding the non-significant complete serial indirect association, this analysis was constrained by the short-term measurement window and was based only on the tragic narrative subsample (*n =* 107), which provided relatively limited statistical power. In addition, the low internal consistency of the narrative transportation scale (*α* = 0.505) may have reduced the precision of the estimates in this exploratory pattern. The small standard deviation of narrative transportation in the tragic subsample also suggests restricted variance, which may have limited its statistical performance in the exploratory analyses.

### 4.4 Theoretical contributions

The present study offers three theoretical contributions. First, the study highlights the mixed emotional structure of responses to tragic narratives. Existing research has typically examined sadness or being moved separately, rarely comparing their relative statistical associations within a single model. By simultaneously entering post-exposure sadness and post-exposure being moved into the same regression model and further conducting dominance analysis, the present study showed that both were oppositely associated with self-reported state resilience in the primary model, and that post-exposure sadness showed a larger model-specific incremental contribution to explained variance than post-exposure being moved. This suggests that the self-reported correlates of tragic narrative exposure cannot be reduced to a single negative emotional response but should be understood in relation to multiple co-occurring emotional components.

Second, the study provides a preliminary empirical basis for cautiously discussing the experience of being moved in relation to broaden-and-build theory within acute negative contexts. The broaden-and-build theory emphasizes the cumulative role of positive emotions in broadening thought-action repertoires and supporting adaptive resources over time. In contrast, the present results suggest that the positive association of being moved with post-exposure state resilience was smaller and less robust than the negative association of sadness within the acute measurement window. This does not refute the broaden-and-build theory, but it suggests that any positive association of being moved with self-reported state resilience may be less immediately observable in acute negative contexts; whether this reflects a genuine temporal boundary requires longitudinal investigation.

Third, the study provides an empirical case for mixed-emotion research in tragic narrative contexts. The coexistence of sadness and being moved within the same tragic narrative context, and their opposing adjusted associations with self-reported state resilience, suggest that co-occurring emotional components within a single context may not show simple additive associations with self-reported outcomes.

### 4.5 Practical implications

The present findings suggest that tragic narrative materials should not be assumed to be uniformly harmful or beneficial; their relevance for momentary self-reported state resilience may depend on the specific emotional responses reported after exposure. For narrative-based education, art-related programs, and mental health communication, the findings suggest a cautious design consideration: future work should examine whether emotional responses beyond sadness, such as being moved, are associated with different self-reported outcomes when painful content is accompanied by meaning-making or relational cues. More broadly, the potential value of tragic narratives may lie not simply in minimizing negative emotional content, but in examining how narrative structures are associated with relational and meaning-making responses.

Furthermore, the exploratory piecewise analysis suggests only a tentative design implication: clearer information presentation and stronger narrative engagement may be associated with greater self-reported being moved, although future studies would need to test whether such design features are related to longer-term psychological recovery or resilience.

### 4.6 Limitations and future directions

Several limitations should be noted. First, sadness and being moved were measured with single-item ratings. Although this approach reduced participant burden and was suited to a pre-post online design focused on momentary affective intensity, single-item measures cannot capture the multidimensional structure of these emotions, do not permit internal consistency estimation, and may be sensitive to individual differences in how emotion labels are interpreted, especially for the Chinese term “被感动.” Future research should therefore employ multi-item measures, including dedicated instruments such as the Kama Muta Scale for being moved, to strengthen measurement precision and capture the multidimensional structure of this emotional experience.

A related limitation concerns common-method measurement and temporal overlap. Post-exposure sadness, being moved, and state resilience were all self-reported within the same post-exposure session. Therefore, the observed associations may partly reflect shared method variance, transient response tendencies, or semantic overlap between emotional states and self-evaluations of state resilience. Future studies should include temporally separated assessments, behavioral or physiological indicators, and follow-up measures to better distinguish short-term emotional self-evaluation from longer-term resilience-related adaptation.

In addition, the sample was recruited online from university and community populations and may not fully represent broader age, educational, or cultural distributions. The generalizability of these findings should be examined in more diverse samples and under more tightly controlled laboratory conditions.

This study focused on the immediate self-reported correlates of tragic narrative exposure. Given that the broaden-and-build theory emphasizes the cumulative role of positive emotions in broadening thought-action repertoires and supporting adaptive resources over time, future longitudinal research should examine whether the association between being moved and self-reported state resilience changes over time.

Sadness and being moved were not directly manipulated. Although the experimental condition was randomized and baseline variables were controlled, sadness and being moved themselves were measured rather than manipulated. Therefore, the relationships between these emotions and self-reported state resilience should be interpreted as adjusted associations rather than established causal relationships. Future research could more directly manipulate the relative intensity of sadness and being moved—for example, by designing materials that vary systematically in high-sadness/low-being moved, low-sadness/high-being moved, and high-sadness/high-being moved conditions—to more rigorously examine their separate and interactive associations with resilience-related outcomes.

Another limitation concerns the control condition. The documentary control condition was designed to provide a neutral non-tragic comparison, but it was not a fully matched neutral fictional narrative. Therefore, the design cannot fully separate tragic emotional content from narrative structure and genre. The observed emotional differences between the tragic narrative conditions and the documentary control condition should therefore be interpreted as differences between the present stimulus conditions rather than as evidence attributable solely to tragic content.

The reported emotional response profiles of the materials were not validated in a separate pilot study before the main experiment. Future studies should include pilot testing to examine whether the stimuli are associated with the intended reported levels of sadness and being moved and to assess familiarity, processing fluency, and perceived narrative engagement before formal data collection.

The narrative transportation scale (*α* = 0.505) and processing fluency scale (*α* = 0.640) showed low internal consistency. This may be because the original scales were developed for longer narratives, while the present study used brief materials, potentially reducing item discriminability. Online testing environments may also have introduced additional variability. Nevertheless, narrative transportation showed significant associations in the exploratory piecewise analyses, suggesting the measure may still have captured some relevant variance. However, any stronger claim about a serial indirect association requires replication under more optimized measurement conditions. The small standard deviation of narrative transportation in the present sample (SD *=* 0.30) further suggests restricted variance, which may have attenuated its statistical performance in the exploratory analyses.

Additionally, because being moved is a multi-component emotional experience, future research could further differentiate its internal dimensions to provide a more fine-grained account of how being moved is associated with self-reported state resilience in tragic narrative contexts.

## 5 Conclusion

The present study suggests that responses to tragic narratives should not be understood as uniformly negative, but as potentially involving mixed emotional components, including sadness and being moved. In the primary regression model, after controlling for pre-exposure resilience, baseline emotions, and experimental condition, post-exposure sadness was negatively associated with post-exposure state resilience, whereas post-exposure being moved was positively associated with post-exposure state resilience. However, the positive association of being moved was no longer statistically significant when broader positive and negative affect were additionally controlled, indicating that this association should be interpreted cautiously. Dominance analysis further indicated that, within the primary model, post-exposure sadness showed a larger model-specific incremental contribution to explained variance than post-exposure being moved. Exploratory piecewise analyses showed several significant adjacent associations among processing fluency, narrative transportation, being moved, and resilience-change scores, but did not support a complete serial indirect association. Overall, the findings suggest that the same-session self-reported correlates of tragic narrative exposure may be better understood in relation to co-occurring emotional components rather than negative affect alone. Because all focal post-exposure variables were assessed through same-session self-report, these findings should be interpreted as momentary self-evaluative associations rather than as evidence of actual short-term adaptation or change in resilience.

## Data Availability

The raw data supporting the conclusions of this article will be made available by the authors, without undue reservation.

## References

[ref1] Appel M. Gnambs T. Richter T. Green M. C. (2015). The transportation scale–short form (TS–SF). Media Psychol. 18, 243–266. doi: 10.1080/15213269.2014.987400

[ref2] Baumeister R. F. Bratslavsky E. Finkenauer C. Vohs K. D. (2001). Bad is stronger than good. Rev. Gen. Psychol. 5, 323–370. doi: 10.1037/1089-2680.5.4.323

[ref3] Berrios R. Totterdell P. Kellett S. (2015). Eliciting mixed emotions: a meta-analysis comparing models, types, and measures. Front. Psychol. 6:428. doi: 10.3389/fpsyg.2015.00428, 25926805 PMC4397957

[ref4] Bonanno G. A. (2004). Loss, trauma, and human resilience: have we underestimated the human capacity to thrive after extremely aversive events? Am. Psychol. 59, 20–28. doi: 10.1037/0003-066X.59.1.20, 14736317

[ref5] Faul F. Erdfelder E. Buchner A. Lang A.-G. (2009). Statistical power analyses using G*power 3.1: tests for correlation and regression analyses. Behav. Res. Methods 41, 1149–1160. doi: 10.3758/BRM.41.4.1149, 19897823

[ref6] Fiske A. P. Seibt B. Schubert T. (2019). The sudden devotion emotion: Kama muta and the cultural practices whose function is to evoke it. Emot. Rev. 11, 74–86. doi: 10.1177/1754073917723167

[ref7] Fredrickson B. L. (2001). The role of positive emotions in positive psychology: the broaden-and-build theory of positive emotions. Am. Psychol. 56, 218–226. doi: 10.1037/0003-066X.56.3.218, 11315248 PMC3122271

[ref8] Fredrickson B. L. Tugade M. M. Waugh C. E. Larkin G. R. (2003). What good are positive emotions in crises? A prospective study of resilience and emotions following the terrorist attacks on the United States on September 11th, 2001. J. Pers. Soc. Psychol. 84, 365–376. doi: 10.1037/0022-3514.84.2.365, 12585810 PMC2755263

[ref9] Graf L. K. M. Landwehr J. R. (2015). A dual-process perspective on fluency-based aesthetics: the pleasure-interest model of aesthetic liking. Personal. Soc. Psychol. Rev. 19, 395–410. doi: 10.1177/1088868315574978, 25742990

[ref10] Graf L. K. M. Mayer S. Landwehr J. R. (2018). Measuring processing fluency: one versus five items. J. Consum. Psychol. 28, 393–411. doi: 10.1002/jcpy.1021

[ref11] Green M. C. Brock T. C. (2000). The role of transportation in the persuasiveness of public narratives. J. Pers. Soc. Psychol. 79, 701–721. doi: 10.1037/0022-3514.79.5.701, 11079236

[ref12] Gross J. J. (1998). The emerging field of emotion regulation: an integrative review. Rev. Gen. Psychol. 2, 271–299. doi: 10.1037/1089-2680.2.3.271

[ref13] Gross J. J. (2015). Emotion regulation: current status and future prospects. Psychol. Inq. 26, 1–26. doi: 10.1080/1047840X.2014.940781

[ref14] Hanich J. Wagner V. Shah M. Jacobsen T. Menninghaus W. (2014). Why we like to watch sad films. The pleasure of being moved in aesthetic experiences. Psychol. Aesthet. Creat. Arts 8, 130–143. doi: 10.1037/a0035690

[ref15] Hiew C. C. (2002). “Measurement of resilience in adults and children,” in Proceedings of the 110th Annual Convention of the American Psychological Association, (Chicago, IL).

[ref16] Hobfoll S. E. (1989). Conservation of resources: a new attempt at conceptualizing stress. Am. Psychol. 44, 513–524. doi: 10.1037/0003-066X.44.3.513, 2648906

[ref17] Larsen R. J. Fredrickson B. L. (1999). “Measurement issues in emotion research,” in Well-Being: The Foundations of Hedonic Psychology, eds. Kahneman D. Diener E. Schwarz N. (New York, NY: Russell Sage Foundation), 40–60.

[ref18] Larsen J. T. McGraw A. P. (2011). Further evidence for mixed emotions. J. Pers. Soc. Psychol. 100, 1095–1110. doi: 10.1037/a0021846, 21219075

[ref19] Luthar S. S. Cicchetti D. Becker B. (2000). The construct of resilience: a critical evaluation and guidelines for future work. Child Dev. 71, 543–562. doi: 10.1111/1467-8624.00164, 10953923 PMC1885202

[ref20] Masten A. S. (2001). Ordinary magic: resilience processes in development. Am. Psychol. 56, 227–238. doi: 10.1037/0003-066X.56.3.227, 11315249

[ref21] Menninghaus W. Wagner V. Hanich J. Wassiliwizky E. Kuehnast M. Jacobsen T. (2015). Towards a psychological construct of being moved. PLoS One 10:e0128451. doi: 10.1371/journal.pone.0128451, 26042816 PMC4456364

[ref22] Oliver M. B. Bartsch A. (2010). Appreciation as audience response: exploring entertainment gratifications beyond hedonism. Hum. Commun. Res. 36, 53–81. doi: 10.1111/j.1468-2958.2009.01368.x

[ref23] Oliver M. B. Raney A. A. (2011). Entertainment as pleasurable and meaningful: identifying hedonic and eudaimonic motivations for entertainment consumption. J. Commun. 61, 984–1004. doi: 10.1111/j.1460-2466.2011.01585.x

[ref24] Ong A. D. Bergeman C. S. Bisconti T. L. Wallace K. A. (2006). Psychological resilience, positive emotions, and successful adaptation to stress in later life. J. Pers. Soc. Psychol. 91, 730–749. doi: 10.1037/0022-3514.91.4.730, 17014296

[ref25] Reber R. Schwarz N. Winkielman P. (2004). Processing fluency and aesthetic pleasure: is beauty in the perceiver's processing experience? Personal. Soc. Psychol. Rev. 8, 364–382. doi: 10.1207/s15327957pspr0804_3, 15582859

[ref26] Rozin P. Royzman E. B. (2001). Negativity bias, negativity dominance, and contagion. Personal. Soc. Psychol. Rev. 5, 296–320. doi: 10.1207/S15327957PSPR0504_2

[ref27] Seibt B. Schubert T. W. Zickfeld J. H. Fiske A. P. (2017). Interpersonal closeness and morality predict feelings of being moved. Emotion 17, 389–394. doi: 10.1037/emo0000271, 28150953

[ref28] Thompson E. R. (2007). Development and validation of an internationally reliable short-form of the positive and negative affect schedule (PANAS). J. Cross-Cult. Psychol. 38, 227–242. doi: 10.1177/0022022106297301

[ref29] Vaish A. Grossmann T. Woodward A. (2008). Not all emotions are created equal: the negativity bias in social-emotional development. Psychol. Bull. 134, 383–403. doi: 10.1037/0033-2909.134.3.383, 18444702 PMC3652533

[ref30] van Laer T. de Ruyter K. Visconti L. M. Wetzels M. (2014). The extended transportation-imagery model: a meta-analysis of the antecedents and consequences of consumers’ narrative transportation. J. Consum. Res. 40, 797–817. doi: 10.1086/673383

[ref31] Wassiliwizky E. Wagner V. Jacobsen T. Menninghaus W. (2017). Art-elicited chills indicate states of being moved. Psychol. Aesthet. Creat. Arts 12, 216–227. doi: 10.1037/aca0000108, 27371692

[ref32] Zickfeld J. H. Schubert T. W. Seibt B. Blomster J. K. Arriaga P. Basabe N. . (2019). Kama muta: conceptualizing and measuring the experience often labelled being moved across 19 nations and 15 languages. Emotion 19, 402–424. doi: 10.1037/emo0000450, 29888936

